# Synthesis of Plasmonically Active Titanium Nitride Using a Metallic Alloy Buffer Layer Strategy

**DOI:** 10.1021/acsaelm.3c01344

**Published:** 2023-12-13

**Authors:** Arthur F. Lipinski, Christopher W. Lambert, Achyut Maity, William R. Hendren, Paul R. Edwards, Robert W. Martin, Robert M. Bowman

**Affiliations:** † School of Mathematics and Physics, Queen’s University Belfast, Belfast BT7 1NN, U.K.; ‡ Department of Physics, SUPA, University of Strathclyde, Glasgow G4 0NG, U.K.

**Keywords:** thin film deposition, sputtering, alternative transition metal nitrides, titanium nitride (TiN), FOM, surface plasmon, ATR, Kretschmann–Reather (KR) configuration, plasmon coupling, single particle spectroscopy, cathodoluminescence (CL)

## Abstract

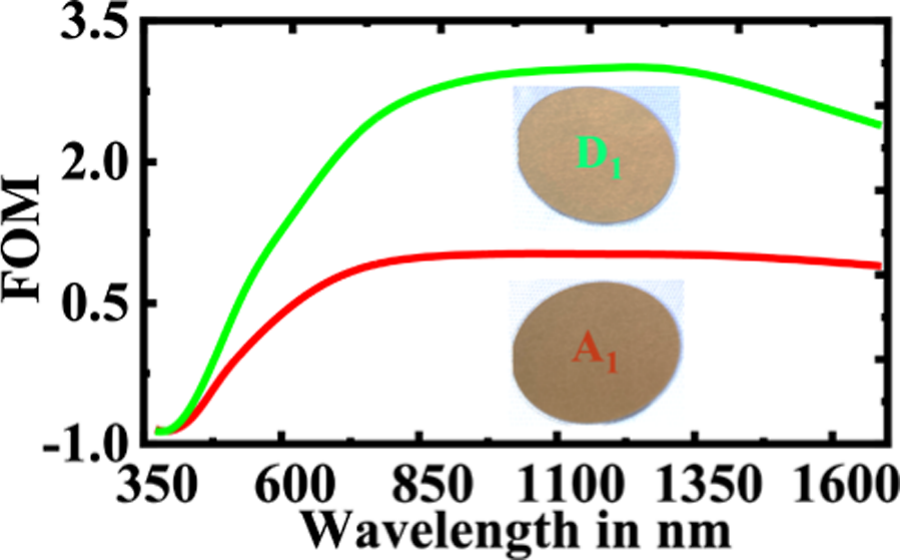

Titanium nitride (TiN) has emerged as a highly promising alternative to traditional plasmonic materials. This study focuses on the inclusion of a Cr_90_Ru_10_ buffer layer between the substrate and thin TiN film, which enables the use of cost-effective, amorphous technical substrates while preserving high film quality. We report best-in-class TiN thin films fabricated on fused silica wafers, achieving a maximum plasmonic figure of merit, −ϵ′/ϵ″, of approximately 2.8, even at a modest wafer temperature of around 300 °C. Furthermore, we delve into the characterization of TiN thin film quality and fabricated TiN triangular nanostructures, employing attenuated total reflectance and cathodoluminescence techniques to highlight their potential applications in surface plasmonics.

## Introduction

Transition metal nitride materials have garnered much recent interest because their plasmonic and refractory properties making them promising candidates for applications which require conditions such as mechanical robustness and high temperatures that limit the use of traditional plasmonic materials like gold or silver.^1^ Recent studies have also demonstrated the surface plasmon-assisted optical response of the metallic TiN in the visible and infrared spectral ranges. The exceptional combination of thermal and chemical stability, which extends even to temperatures as high as 1400 °C, while concurrently exhibiting plasmonic activity,^2^ underscores TiN as an extraordinarily promising candidate for a number of applications, including photothermal-based plasmonic applications, solar absorbers,^3−7^ photothermal medical therapy,^8,9^ and heat-assisted magnetic recording (HAMR) to name a few.^10^ As such, studies have been undertaken to improve the optical properties of TiN to shift plasmonic quality even closer to that of gold.^11−20^

Highly metallic TiN films can be grown by several techniques, such as reactive sputtering, plasma-enhanced atomic layer deposition (PE-ALD),^21,22^ pulsed laser deposition,^6,16,23^ molecular-beam epitaxy,^18,24^ and so forth.

It has been observed that magnetron sputtering provides the potential to deposit smooth and highly metallic TiN thin films, even below 10 nm.^17^ These studies have mostly involved the use of substrates such as MgO and Al_2_O_3_ in order to achieve the best possible lattice matching, which encourages selective crystalline growth.^18,20,21,24−29^ For quite some time, MgO has been the substrate of choice for growing highly textured/epitaxial TiN thin films to yield metallicity and improved optical properties. MgO substrates are comparatively expensive ($/cm^2^), hygroscopic, and harder to integrate into the existing manufacturing processes.^30^ Usually the fabrication process takes place at very high substrate temperatures, typically at 500°–600 °C or even higher, enabling the lattice matching between the TiN film and the substrate.^18,20,24,26−28^ Though high-temperature depositions of TiN on expensive MgO and Al_2_O_3_ substrates have shown excellent performance, they are unlikely to be practical for use in industrial processes. Applications including silicon microelectronics or flexible electronics, especially in complementary metal-oxide-semiconductor technology, require a low ceiling temperature no higher than 500 °C.^31^

In order to qualify for practical or industrial realization, it is necessary to produce TiN within a reduced thermal budget (lower substrate temperatures and no post annealing) and include amorphous technical substrates, such as fused silica, which offers little in the way of orientated film growth. In this context, the use of an additional buffer layer might be an excellent alternative option to fabricate highly metallic TiN films with a high figure of merit (FOM) (−ϵ′/ϵ″, where ϵ′ and ϵ″ are the real and imaginary part of the dielectric function, respectively) at relatively low temperature. For instance, in a study conducted by Ding et al.,^32^ it was demonstrated that the use of a MgO buffer layer on a Si(001) substrate could improve the FOM of TiN produced through PE-ALD, even at a moderate substrate temperature of 450 °C. However, there is a preference for exploring simpler buffer layer options due to their potential attractiveness.

In this context, Cr^33^ or Cr-alloy materials^34^ can be an alternative solution owing to their strength and ductility at higher temperatures.^35^ The use of a Cr as an adhesion/seed layer to deposit metal thin films has been a long-standing standard.^36,37^ The compatibility of Cr materials is already well established^36−38^ while the research on the compatibility of the Cr-alloys, such as Cr_90_Ru_10_, is currently very rare.^39^ Although Dong et al.^39^ have shown that the use of CrRu as an intermediate layer initiates the textured growth of TiN; however, the extensive optical properties of deposited TiN thin films using CrRu as a buffer/seed layer have not been studied so far. Beneficially CrRu does not introduce additional contamination through oxidation at higher temperature deposition.^40,41^

In this article, we have addressed the novel use of a chromium ruthenium (Cr_90_Ru_10_) alloy buffer layer to attain best-in-class FOM at wafer deposition temperatures ∼300 °C and demonstrated the plasmonic functionality in patterned nanoentities. The crystalline characteristics and thickness of the films were investigated by X-ray diffraction (XRD) and reflection techniques. The optical dielectric function of the TiN films was estimated through the experimentally acquired spectroscopic ellipsometry data. We demonstrate the excitation of the surface plasmon polariton (SPP), through its coupling with an evanescent wave of the incident light wave at the TiN/air interface with attenuated total reflection (ATR) using the Kretschmann–Raether configuration (KR configuration). The multilayer reflection model for stratified media was used to support the experimentally acquired data and to demonstrate the sensitivity of the coupling efficiency on mediums of differing refractive index, which may have potential use in the design of refractive index sensors and biosensors. We have also demonstrated the localized surface plasmon-assisted optical properties of a TiN triangular nano-object using hyperspectral cathodoluminescence (CL) imaging and 3D finite difference time domain simulations.

## Experimental Methods

### Titanium Nitride (TiN) Film Deposition

Thin films of TiN with a Cr_90_Ru_10_ buffer layer were deposited by reactive DC magnetron sputtering from a 99.95% pure Ti target and Cr_90_Ru_10_ alloy target in an atmosphere of Ar and N_2_ onto 3 in. fused silica or silicon substrates. The seed layer thickness is chosen to be 25 nm to avoid substrate-based oxidization through the diffusion of oxygen in fused silica.^40,41^ In most cases, the TiN thickness was 50 nm, and that of the Cr_90_Ru_10_ seed was 25 nm, except for films intended for ATR studies where the thicknesses were necessarily reduced. Process parameters were deposition rates, controlled by magnetron power, the partial pressures of Ar and N_2_ mediated through mass flow rates, and substrate temperature. The base pressure was 5 × 10^–9^ Torr and typical process pressure provided by an Ar flow rate of 5 sccm corresponded to 0.75 × 10^–4^ Torr. Optimal flow rates for N_2_ were between 0.8 and 1 sccm and an estimated partial pressure of 0.9 × 10^–4^ Torr for 1 sccm N_2_ and 0.8 × 10^–4^ Torr for 0.8 sccm N_2_. Substrate temperature was independently calibrated by using a thermocouple attached to a test wafer. Deposition rate calibrations, film thickness measurements, and structural characterization were carried out using XRR and XRD on a Bruker D8 Discover X-ray diffractometer.

Crystallite size of thin TiN films was determined by using the Debye–Scherrer equation. , where λ is the wavelength of the X-rays, β is the full width at half maximum of the peak, and θ is the diffraction angle at which the peak is located. Please note that we have not found any existence of the (200) peak at room temperature with no buffer configuration.

### Optical Characterization

The films were characterized optically and plasmonically by ellipsometry and ATR measurements. Optical properties, expressed as dielectric permittivity (ϵ′ and ϵ″), refractive index, and reflectivity, were obtained via a J.A. Woollam M-2000 ellipsometer. Thickness measured via XRR was introduced into the ellipsometry B-Spline model while maintaining the Kramers–Kronig relation.^32^

ATR measurements were done in the Kretschmann–Raether configuration, θ – 2θ, which is widely utilized for its precision in probing surface plasmon behavior. P-polarized (TM) light was obtained using a half-wave plate with a wavelength of 1550 nm was used to excite the SPP at the film/air interface through the substrate using a fused silica equilateral prism placed against the substrate and matched optically using a suitable index-matching fluid (*n*_prism_ = 1.444). The angle of incidence was swept through the critical angle of the prism, with a step size of 0.05° to allow evanescent coupling into the SPP resulting in a minimum in reflectance. The internal angle of incidence within a prism was calculated using Snell’s law to correct it for the light refraction. The power of the incident laser was meticulously adjusted to ensure that neither the thin metallic sample nor the detectors were overwhelmed by the high incident light power (>100 μ W).^42,43^

### Nanostructure Fabrication and CL Studies

The fabrication of TiN nanostructures was carried out by focused ion beam (FIB) (LYRA3 FIB–SEM, Tescan) on a 50 nm TiN film with 25 nm Cr_90_Ru_10_ seed layer deposited on a Si substrate at 300 °C. Si was needed for CL experiments as it does not emit in the spectral range of 450–850 nm and is sufficiently conductive to prevent the electron beam charging effect. The dielectric function of TiN on top of the Si substrate at 1.0 sccm condition at a substrate temperature of 300 °C is plotted in Supporting Information (Figure S1). The nanostructures were milled in the TiN layer by using a high-energy gallium ion beam. The beam energy and the ion beam current were kept at 30 keV and 30 pA, respectively.

CL experiments (shown in Figure S2) were conducted on an FEI Quanta 250 FEG scanning electron microscope in the high vacuum mode. The CL data were collected in the hyperspectral imaging mode where each pixel of the image contains a full set of spectral information during the scan over the region of interest.^44^ An electron beam of 30 keV energy (current ∼6.6 nA) is incident onto the sample, and emitted photons caused by the interaction of electron energy and the particle’s plasmon mode are directed to the cooled EMCCD with 1600 channels and 16 μm pixel pitch through the light collection path (in order): reflecting objective; fused silica vacuum window; off-axis parabolic mirror; and spectrograph as shown in Figure S2 (1/8 m spectrograph with 400 L/mm grating blazed at 500 nm and centered at 450 nm). However, none of these should have a pronounced feature in the range of 480–700 nm. The sample was mounted on the SEM sample holder and tilted 10° toward the CL collector for maximum light collection.^44^

### COMSOL Multiphysics

The TM polarization-based reflectance properties of TiN thin films were calculated using a commercial finite element full wave solver (COMSOL Multiphysics).^45^ The electromagnetic wave propagation is governed by Maxwell’s wave equation in the frequency domain.

An active port boundary condition was used on the top side to simulate the incident light whereas a passive port was used at the bottom to minimize any artifact caused by the reflection in the computational domain. Floquet boundary conditions were also imposed on other boundaries to enable the symmetry of the electric field. The wavelength-dependent dielectric constants for TiN and Cr_90_Ru_10_ were taken from ellipsometry measurements. Those measurements fully agree with the analytical calculations of the TiN response and further validate the numerical analysis and support the experimental results as evident in Figure S3.

### FDTD Simulations

Commercially available 3D finite-difference time-domain (FDTD) numerical simulations, specifically Ansys FDTD, were employed for the 3D-FDTD simulations. In FDTD, the Maxwell’s equations are solved in the discretized space and time domains using the Yee algorithm.^46^ The electron beam was modeled as a series of closely spaced dipoles having a phase difference related with the electron velocity. The current density is given by:
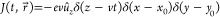
1where *e* and *v* represent the electron charge and the velocity of the electron (*v* = 0.32*c* corresponding to 30 keV electron energy), respectively. The electron beam is moving down along *z* (unit vector is denoted as ) direction and initial coordinate of the e-beam was set at (*x*_0_, *y*_0_). The phase factor is defined as (*z*/*v*). The edge length and the thickness of the modeled triangular nanostructure were set as 1000 and 50 nm, respectively. The material property of the TiN (on a Si substrate) was defined by the refractive index information measured by ellipsometry (shown in Figure S1). We have calculated the Poynting vector components along the normal direction (*P*_*z*_) at a large distance away from the sample in the upper hemisphere.

The Si substrate was set to have a refractive index of 4,^47,48^ with a dimension of 5 μm × 5 μm × 2 μm. A mesh override region (3 nm × 3 nm × 2 nm) all over the TiN nanostructure along with the auto nonuniform meshing having mesh accuracy of 3 were used to calculate the CL response accurately. A very detailed description about the CL response from TiN nanoparticles can be found elsewhere.^47,48^

## Results and Discussion

**Figure 1:**
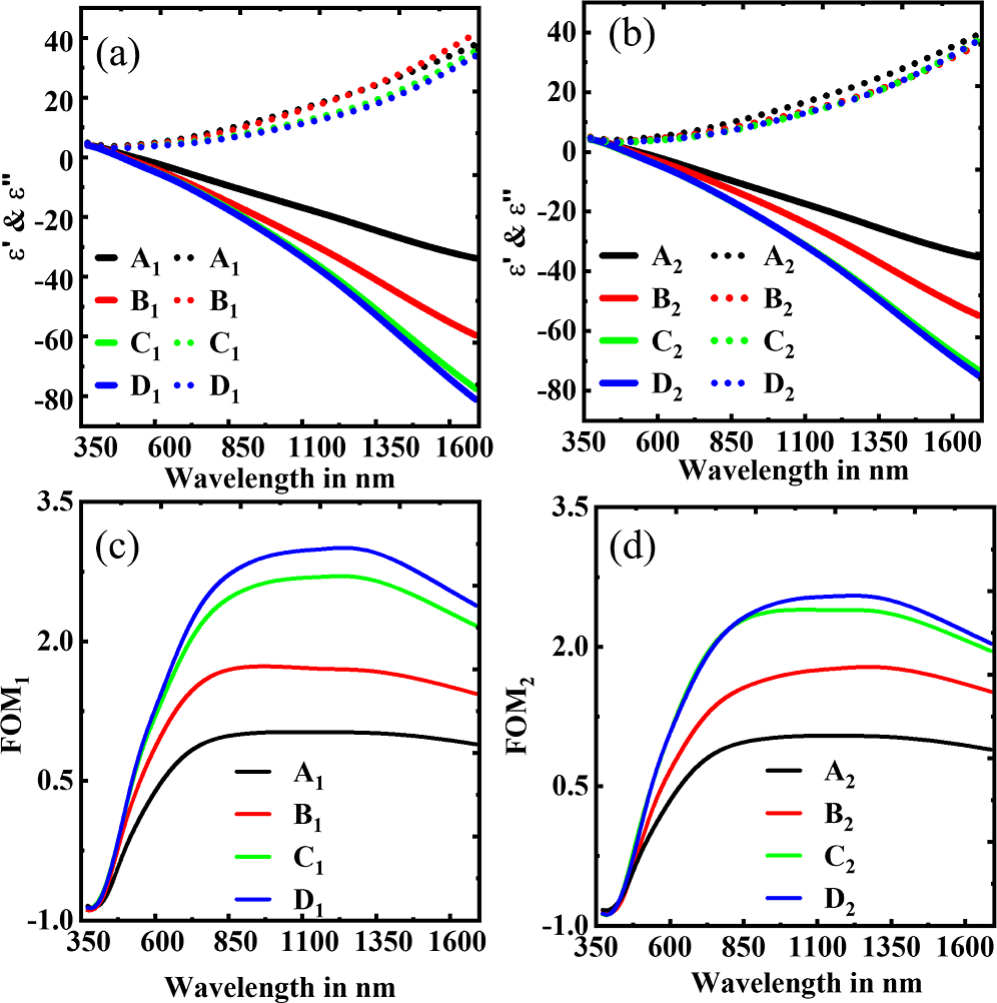
Dielectric constants of the different TiN thin film samples with Cr_90_Ru_10_ buffer layer deposited at (a) *P*_1_ and (b) *P*_2_. The solid lines in both (a,b) represent the real part of the dielectric function while dotted lines represent the imaginary part. (c,d) show respective FOMs.

Table 1 shows the deposition conditions of 50 nm films on top of a 25 nm Cr_90_Ru_10_ buffer layer on fused silica wafers (cross-sectional TEM image as shown in Figure S4). Figure 1 shows the permittivities of the films. It is very clear from both Figure 1a,b that the real part of permittivity shows more metallic behavior with increasing temperature for both partial pressures. Moreover, the optical losses are reduced significantly with elevated substrate temperatures as shown in Figure 1a,b. Although the trend is common for both partial pressures, the relative change in the optical losses in TiN thin films at different wafer/substrate temperatures is more prominent with a lower nitrogen partial pressure.

**Table 1:** Deposition Conditions of all the TiN Thin Films Studied Here

sample	substrate temperature in °C	partial pressure (Torr)
A_1_	room temperature (RT)	0.8 × 10^–4^ (*P*_1_)
A_2_	RT	0.9 × 10^–4^ (*P*_2_)
B_1_	150	0.8 × 10^–4^ (*P*_1_)
B_2_	150	0.9 × 10^–4^ (*P*_2_)
C_1_	225	0.8 × 10^–4^ (*P*_1_)
C_2_	225	0.9 × 10^–4^ (*P*_2_)
D_1_	300	0.8 × 10^–4^ (*P*_1_)
D_2_	300	0.9 × 10^–4^ (*P*_2_)

Figure 1c,d represents the FOM of the TiN samples. It is clear from both the figures that the samples C_1_, D_1_, C_2_, and D_2_ are superior to any of those deposited below 150 °C (A_1_, B_1_, A_2_, and B_2_) for both partial pressures. The FOM reaches a maximum at 2.8 for the D_1_ sample, whereas C_1_, C_2_, and D_2_ reach around 2.65. It is worth noting that the films C_1_, D_1_, C_2_, and D_2_ show comparable FOM numbers to those films deposited at very high substrate temperature (above 500 °C) using reactive DC magnetron sputtering.^11,17,49,50^

After optimization, we have found that changes in both the real and imaginary parts were very marginal for the samples C_1_, D_1_, C_2_, and D_2_.

**Figure 2:**
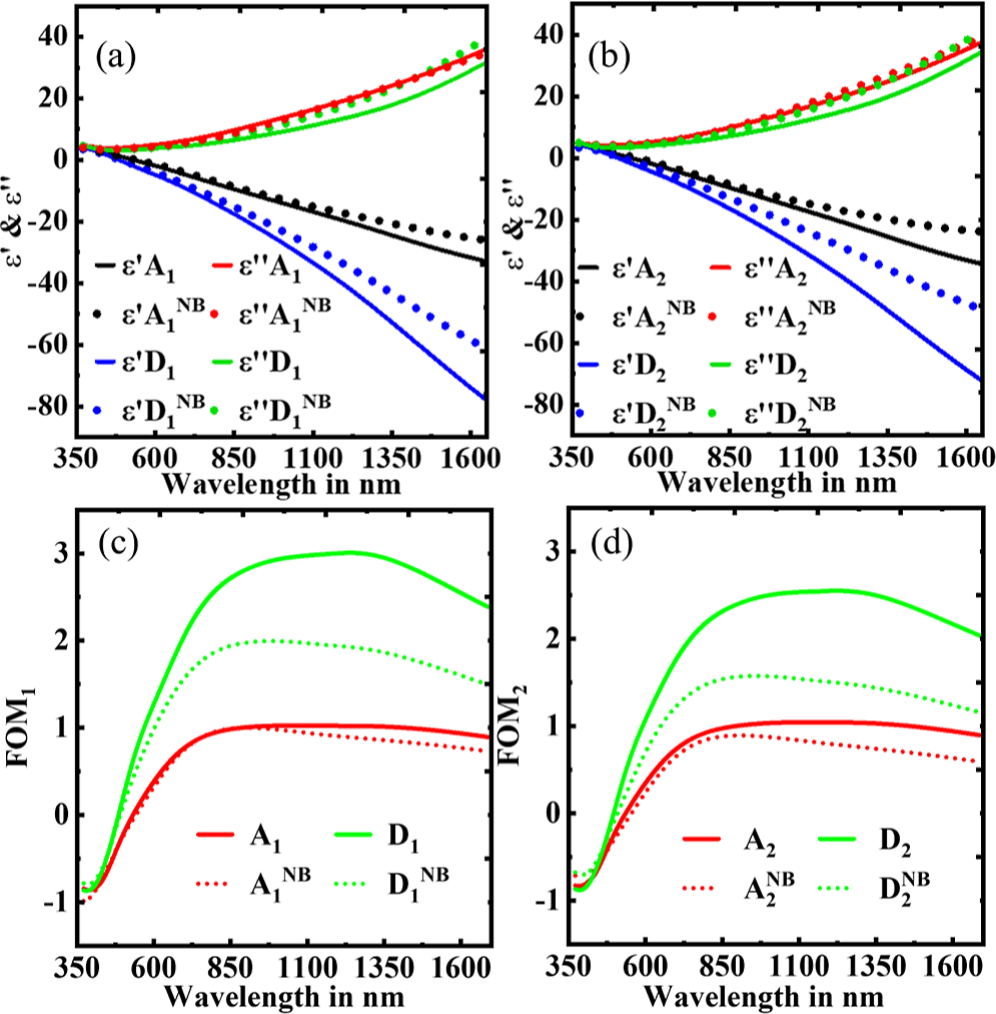
Comparison of dielectric function between TiN thin films with a buffer and no buffer (NB) layer deposited at RT and high temperatures at both *P*_1_ and *P*_2_. (a) A_1_ and D_1_ and (b) A_2_ and D_2_ and their respective FOMs (c) for figure (a) and (d) for figure (b).

To gain a deeper insight into the enhancements shown in Figure 1, we compared the dielectric functions of the samples with and without buffer (NB) along with the RT results in Figure 2. It is very clear from Figure 2a that sample D_1_ has the best optical properties compared to the high temperature/NB TiN thin film at the same temperature (D_1_^NB^) and the film deposited at RT (A_1_). The trend is also consistent for sample D_2_, grown at the higher rate of N_2_ flow (1.0 sccm) as shown in Figure 2b. Their respective FOMs are shown in Figure 2, where FOM (c) is *P*_1_ and FOM (d) is *P*_2_. The optical properties of all deposited samples at different temperatures and partial pressures are shown in Supporting Information, Figure S5.

**Figure 3:**
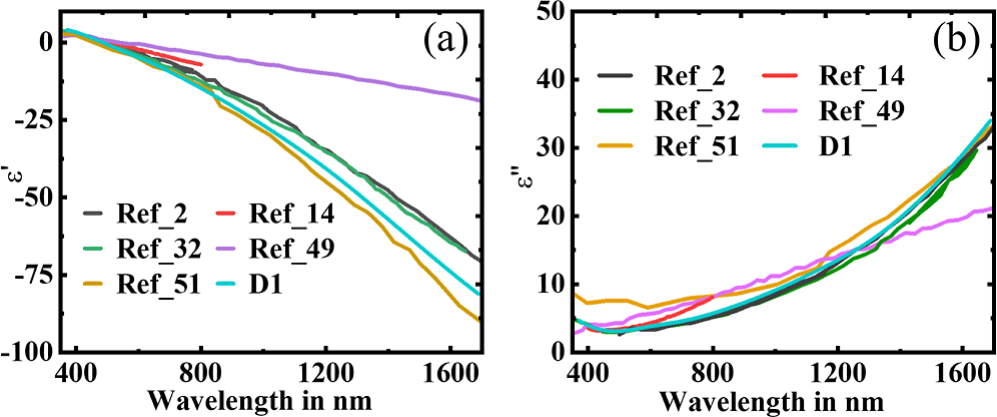
Comparison of (a) real and (b) imaginary parts of sample D_1_ with respect to the other published studies. Reproduced with permission from refs (2) and (51). Copyright 2021 John Wiley and Sons and Copyright 2018 American Vacuum Society, respectively.

The dielectric functions of D_1_ thin film have been compared with those from prior studies (as shown in Figure 3a,b), using similar methodology but different deposition conditions as shown in Table 2 below.^2,14,32,49,51^

**Table 2:** Reference Sample Summary

references	substrate	interlayer	temperature (°C)
(2)	Al_2_O_3_(0001)	N/A	400
(14)	Al_2_O_3_	N/A	250
(32)	Si(001)	MgO	450
(49)	Al_2_O_3_	N/A	800
(51)	Al_2_O_3_(0001)	N/A	400

Although Smith et al.^51^ have reported that the use of a sapphire substrate may lead to a better response, with a substrate temperature in the range of 300–400 °C. This method also suffers from the poor cost-effectiveness of the sapphire substrate. It is also important to note that our results offer improved optical responses compared to the results reported by Ding et al.,^32^ where the TiN was fabricated using MgO as the buffer layer. The use of a MgO layer may improve the lossy characteristic of the TiN thin film (as shown in Figure 3b) due to better lattice matching.^24^ It also suffers additional oxidization^32^ and poor cost-effectiveness in industrial processes. As discussed earlier, the utilization of Cr_90_Ru_10_ on amorphous substrates plays a crucial role in the deposition of plasmonically active TiN.

**Figure 4:**
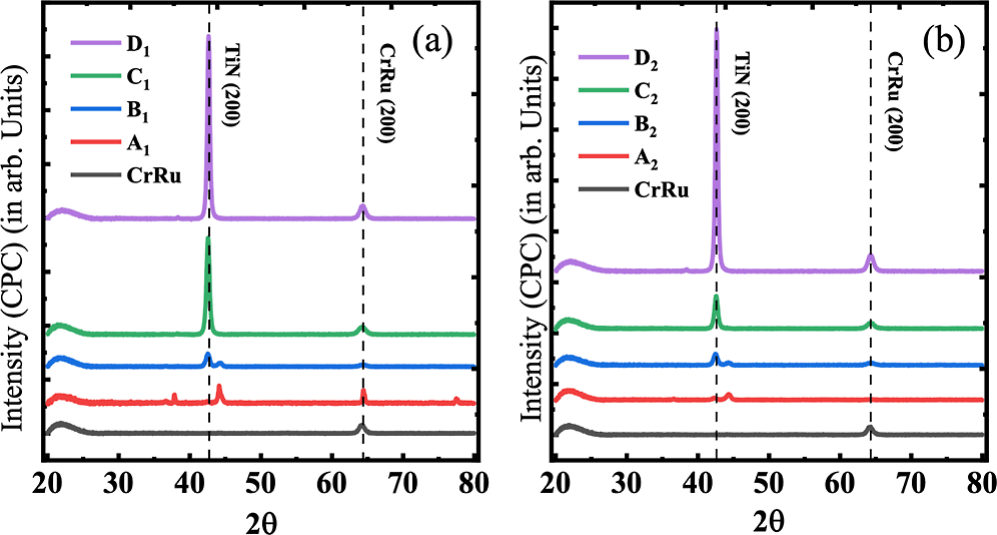
XRD scans of TiN samples deposited at (a) 0.8 × 10^–4^ (*P*_1_ Torr) and (b) 0.9 × 10^–4^ Torr (*P*_2_ Torr).

The θ–2θ XRD pattern plots for all of the deposited films are shown in Figure 4. The presence of the buffer layer initiates the growth of TiN thin films in the preferred (200) direction, predominantly at ∼42.5°.^52,53^ The monotonically increasing peak intensity with increasing substrate temperature, shown in Figure 4a,b (for both *P*_1_ and *P*_2_, respectively), suggests a homogeneous/uniform and textured fabrication of TiN thin films. Interestingly, the growth of TiN along the plane having least surface energy, that is (200), also suggests the formation of TiN films with fewer film defects and growth strain.^54^ The Cr_90_Ru_10_ (200) peak is indexed around ∼64°.^39^ The presence of smaller peaks around the central TiN peak can be attributed to the formation of oxide. In comparison with the XRD plots of TiN thin films with NB layer conditions (Figure S6), we have observed that the presence of Cr_90_Ru_10_ buffer layers plays an important role in the preferential growth of all TiN thin films along (200) direction. However, it is very clear from the X-ray analysis that the deposited TiN films are nonepitaxial^16^ but highly textured^11^ in nature.

Table 3 shows the comparison of crystallite size/grain size of TiN thin films with and without a buffer layer. It is noticed that the higher temperature depositions in the presence of the Cr_90_Ru_10_ buffer layer not only initiate the preferential textured growth along (200) but also lead to the formation of TiN films having larger crystallite size/grain size, which can be attributed to the improvement of the loss function in the films, reducing carrier scattering.^16,55^

**Table 3:** Crystallite Size of TiN Thin Films^a^

	substrate temperature in °C			
grain sizes in nm	RT	150	225	300
grain size at *P*_1_ with buffer/NB	17.5/–	15.7/14.4	19.0/16.8	22.7/17.0
grain size at *P*_2_ with buffer/NB	18.4/–	14.5/12.9	17.4/15.7	23.0/19.9

a As shown in the XRD graphs with NB layer in Figure S6, the intensity of the (200) is very low; thus, the Debye Scherrer evaluation is not possible.

High-temperature deposition is always favorable for better thin film growth as it contributes toward minimizing the structural defects.^11,51,56^ This trend is consistent with our present study as shown in Figures 1 and 2. Both of the responses in the real and imaginary parts are significantly improved in the presence of a buffer layer, characterized by lower real and imaginary parts of the dielectric permittivity. The Cr_90_Ru_10_ has a cubic lattice structure along the [200] direction (Figure S7), that plays an important role in improving the optical response of the TiN thin films by minimizing the lattice mismatch factor^17,32,50,51^ along with maintaining the cube-on-cube growth factor.^23,32^ The improvement in the optical losses can be attributed in different ways: either the buffer layer of Cr_90_Ru_10_ improves the crystallinity^32^ or the compressive strain of the TiN film^51,57,58^ on Cr_90_Ru_10_ plays a crucial role at different substrate temperatures or both. Utilizing a buffer layer could offer advantages in creating plasmonically active TiN films with enhanced metallic behavior.

### Titanium Nitride Annealing Studies

**Figure 5:**
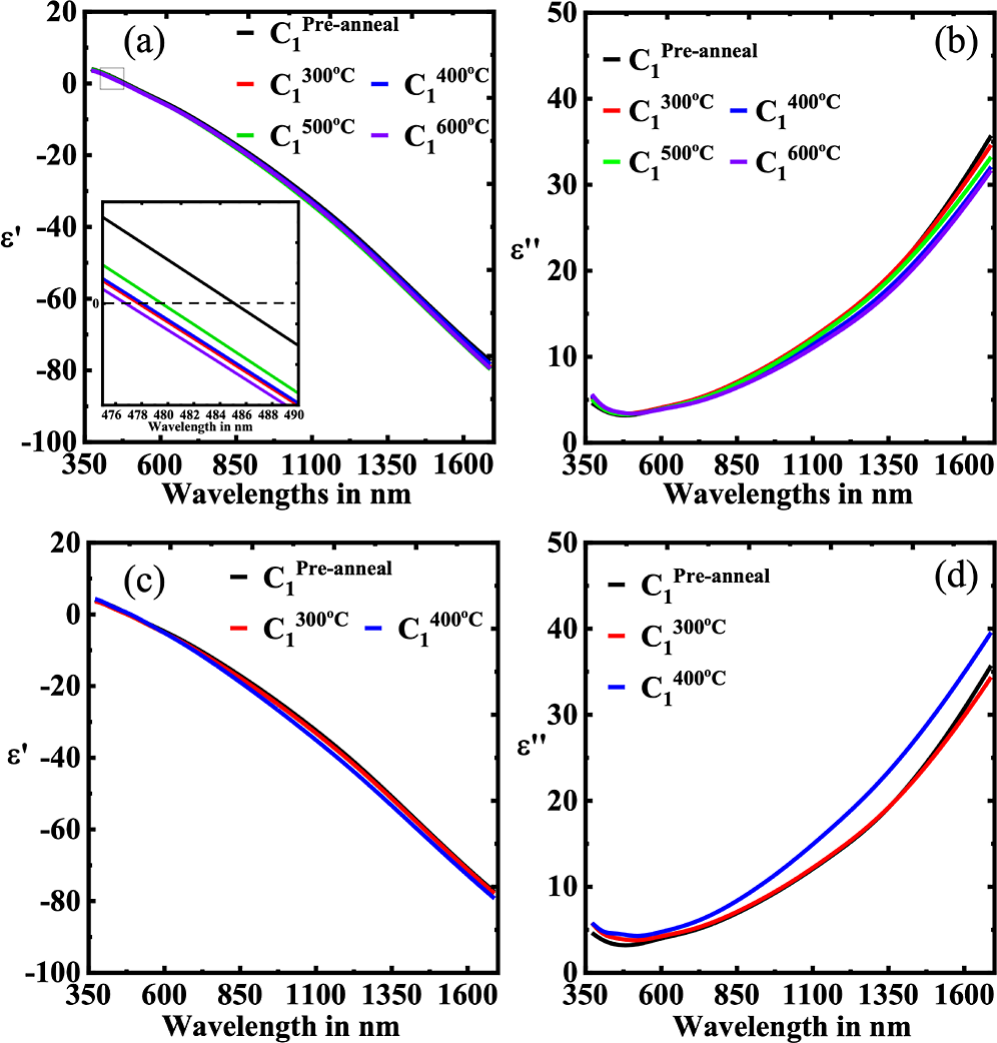
TiN anneal study results. (a,b) Dielectric responses at various temperatures under (a,b) vacuum and (c,d) atmospheric conditions.

To qualify as a potential material for thermoplasmonic applications, thermal robustness must be assessed. To this end, anneals were carried out on thin film sample C_1_, deposited at 225 °C and at *P*_1_. Samples were annealed in the range of 300–600 °C with 100 °C increments in atmospheric and vacuum conditions. Figure 5a,b shows the change in optical response for (C_1_) before and after various vacuum anneals. Both the real and imaginary parts of the dielectric function are improved with higher temperature annealing in vacuum. In addition, the screened plasma frequency (zero crossover) shows a prominent blue-shift trend with increasing temperature due to the bigger crystalline size, as well as enhanced TiN crystallinity.^52,59^ It is also worth mentioning that the change in both the optical responses follows a monotonous trend except the 500 °C result, which might be attributed as an anomalous factor/artifact. Atmospheric anneals of TiN are shown in Figure 5c,d. The real part of the TiN shows a very stable response even under atmospheric conditions. Interestingly, the imaginary part shows a more lossy trend with increasing annealing temperature, which is likely due to the growth of the oxide layer as shown in Table S1. Due to the damage caused by the atmospheric annealing at very high temperatures alongside the 300–400 °C anneals, here it is not possible to fit to the ellipsometry data.

This study shows that the sputtered film is capable of enduring temperatures as high as 600 °C under vacuum conditions. This attribute holds significant potential for various high-temperature applications, including solar thermophotovoltaics^60^ and HAMR,^61^ which could have wide-ranging implications.

### Surface Plasmon-Assisted Optical Properties

#### Attenuated Total Reflectance

**Figure 6:**
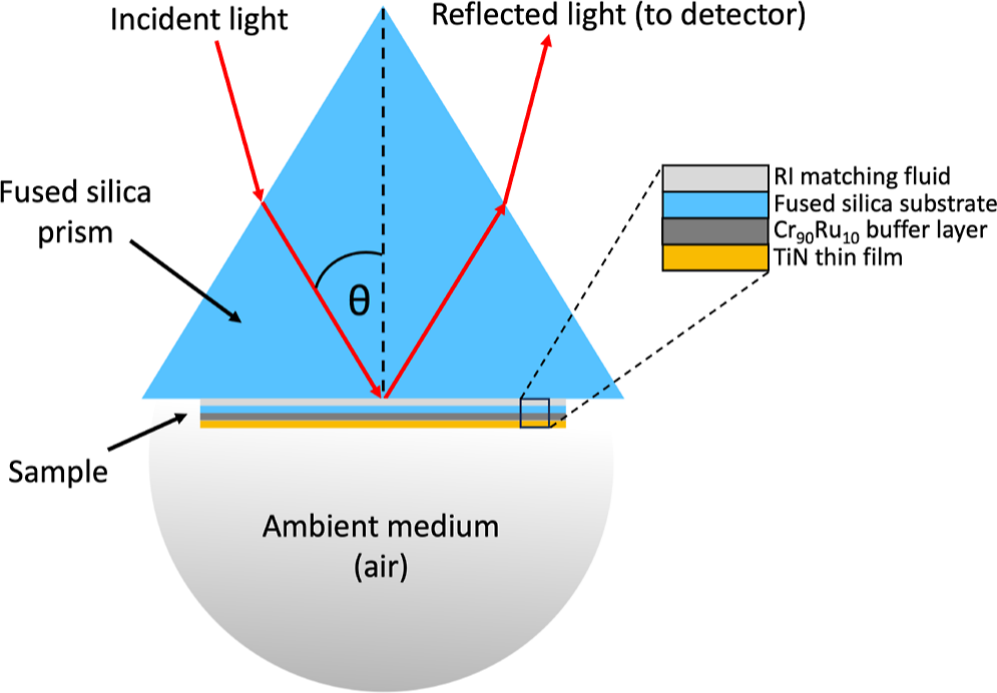
Schematic of the optical coupling setup in the Kretschmann configuration.

This experiment has been performed in Kretschmann configuration, and it is being used to probe the SPR capabilities of the TiN thin films. The incoming light needs to be evanescently coupled through optically lossy Cr_90_Ru_10_ to reach the TiN thin film as shown in Figure 6. In this configuration, a compromise has to be made between the thickness of the buffer layer and the thickness of the TiN to achieve the best results possible. The experimental ATR setup in Kretschmann configuration is shown in Figure S9.

Before performing the experiments, the optimum coupling thickness was theoretically calculated using a multilayer reflection model for stratified media for the highest surface plasmon resonance efficiency.^62−64^

**Figure 7:**
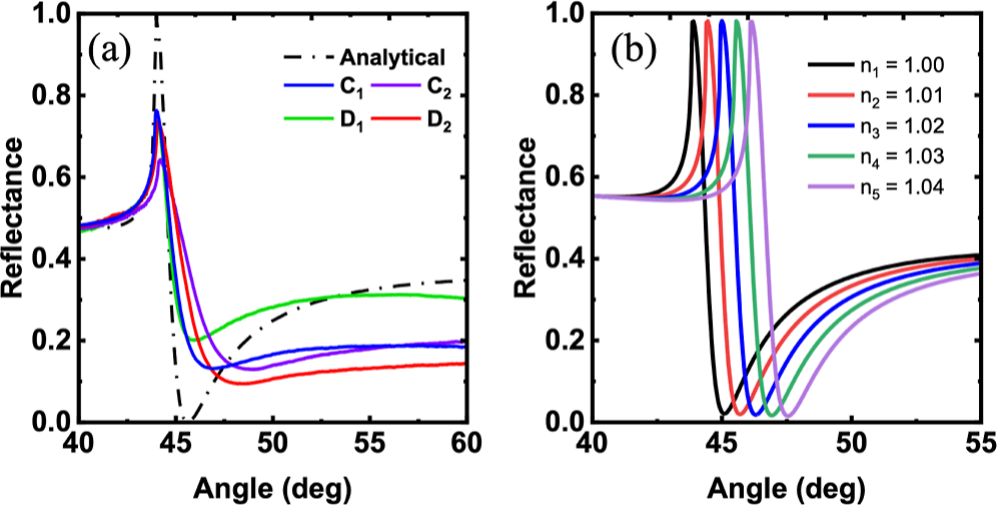
(a) Experimental ATR results of the TiN samples with a buffer layer compared to the analytical data. (b) Analytical ATR results showing the sensitivity of the TiN film to changes in the refractive index of the ambient medium.

It was found that the optimal thickness for both the TiN samples C_1_ and D_2_ was 18 at 1550 nm wavelength. The dip in the reflectance spectra, shown in Figure 7a, relates to the excitation of SPPs at the TiN/air interface.^43,65^ The calculated reflectance spectrum is in good agreement with the experimental responses as shown in Figure 7a. The broadening in all the spectra is very common and it can be attributed to the low carrier mobility (electrons) due to the larger absorptivity of TiN films than conventional plasmonic materials such as Au and Ag.^66^ A slight mismatch between the calculated and experimentally acquired spectra can be understood as follows: a 2 nm seed layer of Cr_90_Ru_10_ is used to fabricate TiN thin films suitable for reflectance measurements. As Cr_90_Ru_10_ is optically absorptive, it may cause such a discrepancy and distort some of the coupling capabilities of TiN. In addition, many factors like temperature, humidity, build up of hydrocarbons on the metal surface, and so forth may have a role during the experimental measurements which cannot be accounted for in the calculated spectrum.

In order to explore the plasmonic behavior of the TiN thin film for sensing purposes, we calculate the reflectance by changing the refractive index of the ambient medium as shown in Figure 7b. In addition, the sensitivity value for the TiN sample curves has been calculated using the following equation:
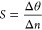
2

The sensitivity of TiN, denoted as *S*_TiN_, has been determined to be approximately 60 theta RIU^–1^. This indicates that TiN exhibits high sensitivity suitable for sensing applications. Even minute changes in the refractive index of the external or ambient medium (whether it is metal or dielectric) can lead to substantial variations in the coupling angle, making TiN an excellent choice for sensing applications.^67,68^

#### CL Analyses

**Figure 8:**
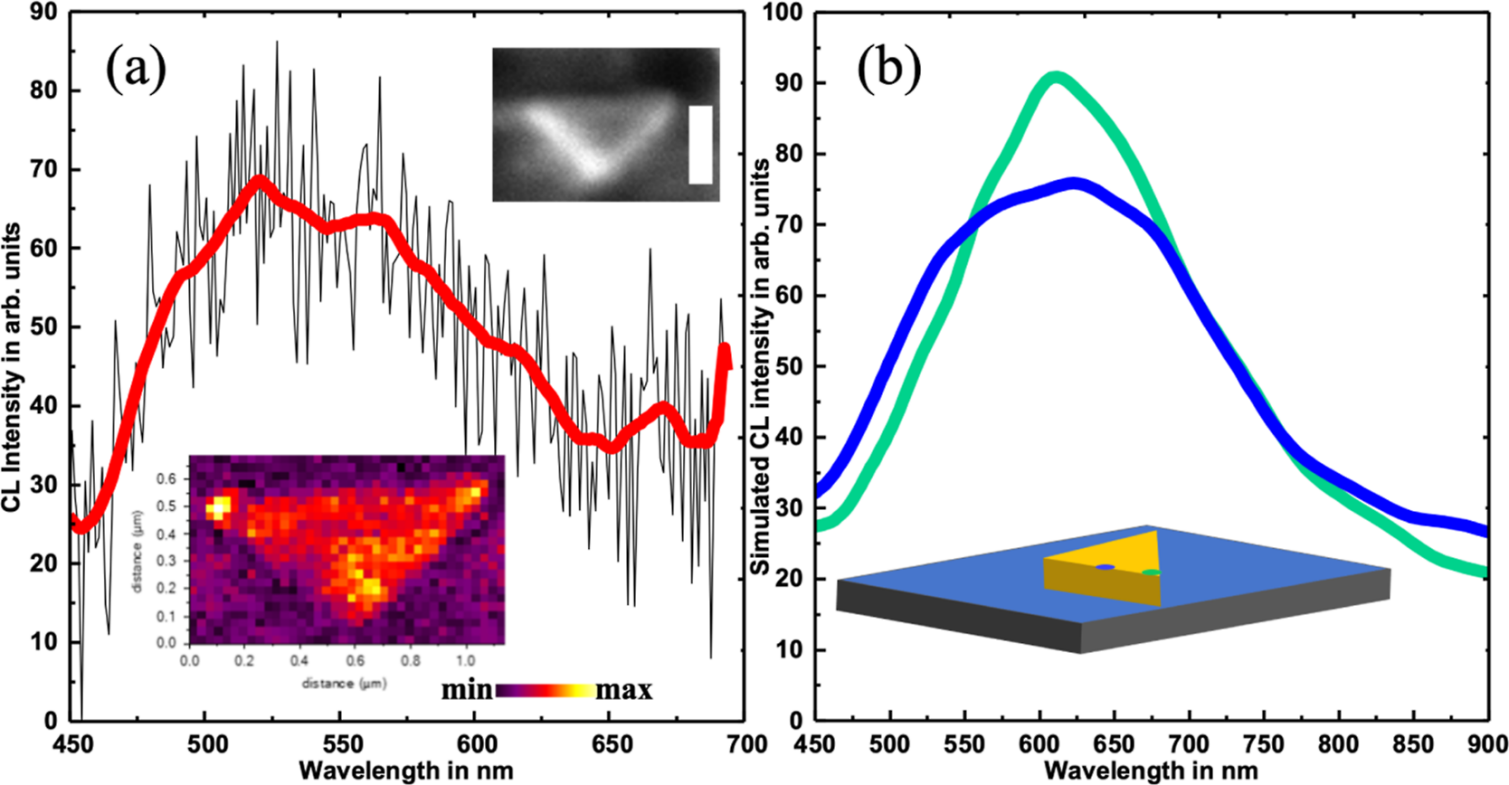
(a) Experimental CL response from triangular nanoparticle. (b) Simulated CL spectra from nanoparticle in the presence of the substrate. The CL spectra were calculated by placing the e-beam at two different locations over the nanoparticle (represented with different colors). The scale bar of the inset SEM image is 500 nm.

The CL response was recorded in the hyperspectral imaging mode. Furthermore, CL spectra were extracted using principal component analysis (PCA) based on the nonlinear iterative partial least-squares (NIPALS) method as described briefly elsewhere.^69^ The calculated scree plot based on the aforementioned technique (shown in Figure S10) suggests the existence of a single PCA component. The first eigenspectrum and the corresponding CL image are shown in Figure 8a. A secondary electron image is also shown in the inset of Figure 8a. It is clear from the CL map (in Figure 8a) that photon emission is very prominent, particularly from the apex regions due to the strong electromagnetic coupling between the incident electron beam and the particle’s plasmon mode.^70^ It is also interesting to note that photon emission is comparatively much less prominent along the edges (particularly the edge on the right side of the image) of the triangular nanoparticle. The surface plasmon resonance is centered around a wavelength of 580 nm. 3D-FDTD simulations were carried out to explore the LSP response in a more detail way. We have modeled the nanotriangle having the edge length of 1000 nm and being placed on a Si substrate. The details of the numerical calculations are given in the method section. The calculated results shown in Figure 8b show very good agreement with the experimentally acquired CL results.

The CL responses were calculated by placing the electron beam at two different places over the modeled nanotriangular particle: at the tip position (tip-excitation) and at middle of the edge (edge-excitation). Figure 8b shows that the plasmon mode associated with the tip-excitation and the edge-excitation is centered around ∼600 and ∼630 nm, respectively. The intensity of the tip excitation is 25% stronger than that of edge-mode excitation. This suggests that there is efficient coupling of the plasmon modes at these apex regions. The spectral mismatch between experimental and simulated data can be attributed to the imperfect modeling of the nanotriangle object in terms of morphological details, including surface roughness,^71^ radius of curvature of the apexes, and so forth. A broadening of ∼200 nm is common in both the experimental and the simulated CL results. Such broadening in CL response from bigger metal particles mainly arises due to the radiation losses.^27,67,72,73^ The oscillation nature of the individual plasmon modes are masked due to the huge broadening in their spectral response compared to the distance between two resonance wavelengths (∼30 nm).^70^ The photon emission or the surface plasmon excitation (shown in Figure 8a) is not related to any pure type of higher order mode of oscillations as the particle is bigger in size (retarded regime). The nature of the surface plasmon response might be the first-harmonic plasmon mode,^72^ mixed,^73^ hybridized, or substrate-modified in nature.^74^ In addition, the presence of the “hotspot” (where the electromagnetic local density of state is relatively high) at the apex regions has great importance particularly in heat-assisted-based modern nanophotonic technologies, like HAMR,^75−78^ cancer therapy,^8,79^ photothermal imaging, and so forth.^20,80^ Moreover, the difference in the intensity of the electron-induced photon emission from different apexes caused by the tilted position of the sample stage is also providing a hint of the directionality of the photon emission.^69^

## Discussion/Conclusions

In summary, we have investigated the role of the Cr_90_Ru_10_ buffer layer in improving the optical properties of a titanium nitride thin film deposited on top of a fused silica substrate. We showed that process-dependent parametrization in terms of substrate temperature and the nitrogen gas flow rate also plays an important role in achieving better metallic quality and lower loss. The postdeposition annealing studies revealed the mechanical/thermal stability of the TiN thin films under vacuum conditions.

Reflectance studies employing the Kretschmann–Raether (K–R) configuration confirmed the successful excitation of SPPs at the air/TiN interface. Simulated reflectance studies, incorporating different refractive indices, highlighted the potential of TiN thin films for surface plasmon-assisted sensing applications. Hyperspectral CL studies, along with simulations, unveiled localized surface plasmon resonances in isolated TiN nanotriangle fabricated using FIB milling. In conclusion, our approach to achieving metallic TiN with minimal loss on industrially relevant substrates, even at substrate temperatures below 300 °C, presents a promising avenue for various plasmonic and metamaterial applications.

## Supporting Information Available

Dielectric function of TiN on the Si substrate; schematic of CL; experimental vs theoretical analysis of SPR curves; TEM–EDX of TiN thin films; dielectric functions of all deposited TiN thin films; XRD of TiN thin films with NB; XRD plot of Cr_90_Ru_10_; oxidization effect after annealing studies; experimental setup of ATR; and scree plot of eigenvalues (PDF)
