# Supplementary material for: Synthesis of Plasmonically Active Titanium Nitride Using a Metallic Alloy Buffer Layer Strategy
Source: ACS Appl Electron Mater. 2023 Dec 13;5(12):6929–37. doi: 10.1021/acsaelm.3c01344 (PMC10753803; doi:10.1021/acsaelm.3c01344)
Supplement: Supplementary file 1 — el3c01344_si_001.pdf [file el3c01344_si_001.pdf]

Supporting Information

Synthesis of Plasmonically Active Titanium Nitride Using a Metallic Alloy Buffer Layer Strategy

Arthur F. Lipinski,[†,§] Christopher W. Lambert,[†,§] Achyut Maity,[*,†,§] William R. Hendren,[†] Paul R. Edwards,[‡] Robert W. Martin,[‡] and Robert M. Bowman [*,†]

† School of Mathematics and Physics, Queen's University Belfast, Belfast BT7 1NN, U.K.
‡Department of Physics, SUPA, University of Strathclyde, Glasgow G4 0NG, U.K.

§Contributed equally

*E-mail: a.maity@qub.ac.uk; r.m.bowman@qub.ac.uk

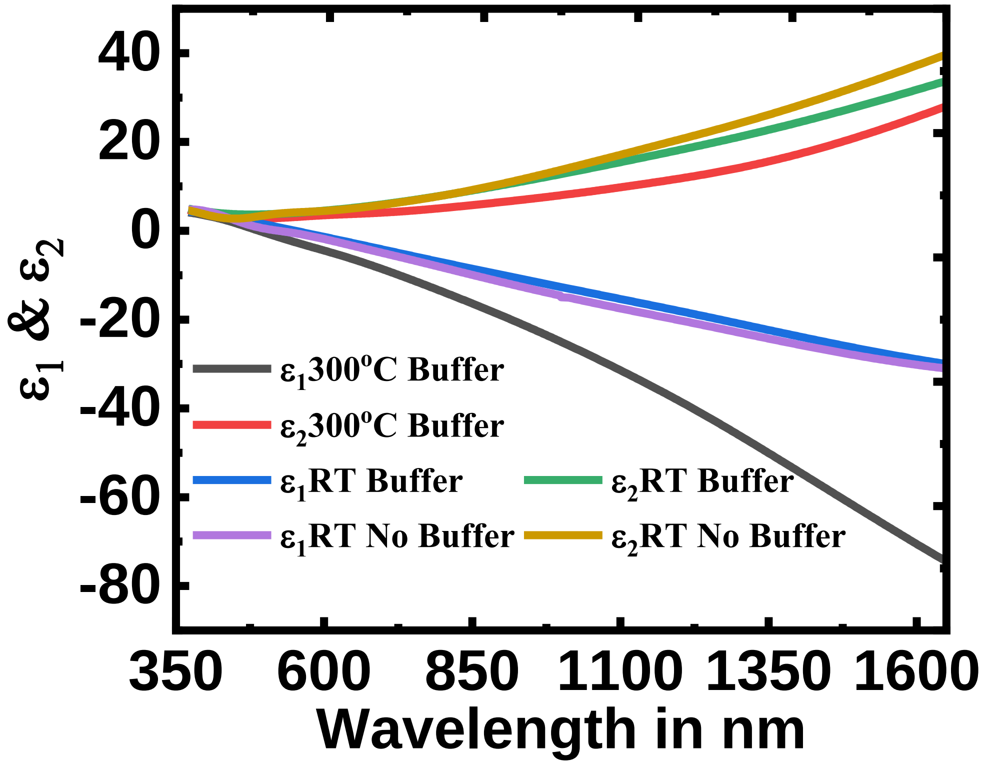

Figure S1: Dielectric function of 50 nm TiN on top of Si wafer using a 25 nm layer of $Cr_{90}Ru_{10}$ as a buffer layer. We have also compared the permittivity of TiN deposited on Si wafer with buffer layer at 300°C with TiN at room temperature buffer and no buffer situation.

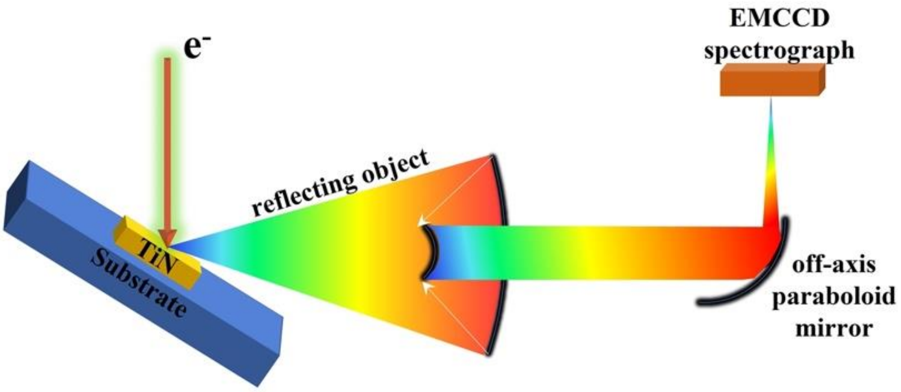

Figure S2: A schematic of cathodoluminescence setup.

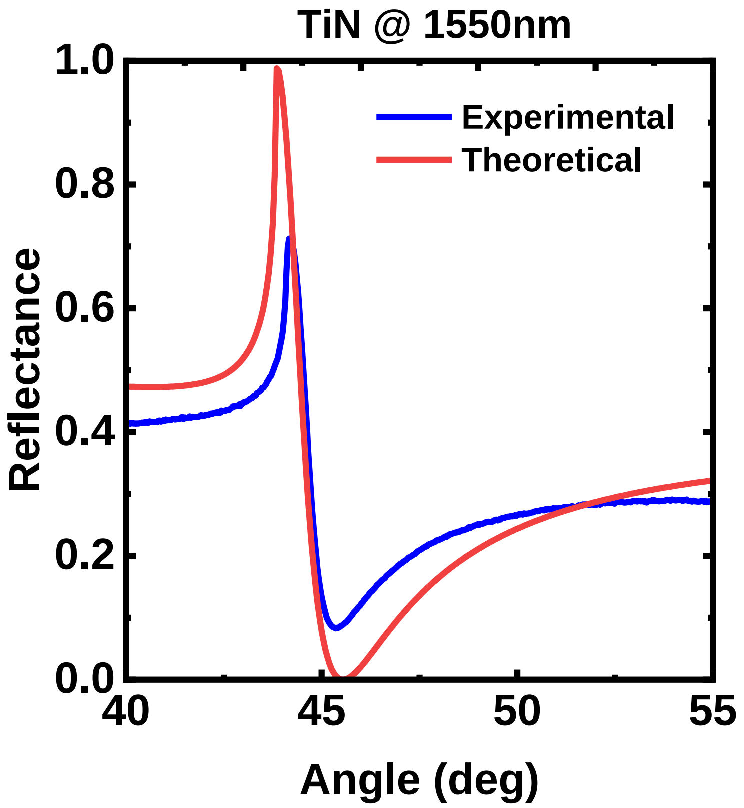

Figure S3: Comparative analysis of SPR reflectance curves acquired from the ATR setup in Kretschmann-Raether configuration, illustrating the optimized TiN sample against the corresponding theoretical curve for TiN. Strong concordance is observed between experimental and COMSOL Multiphysics-simulated results.

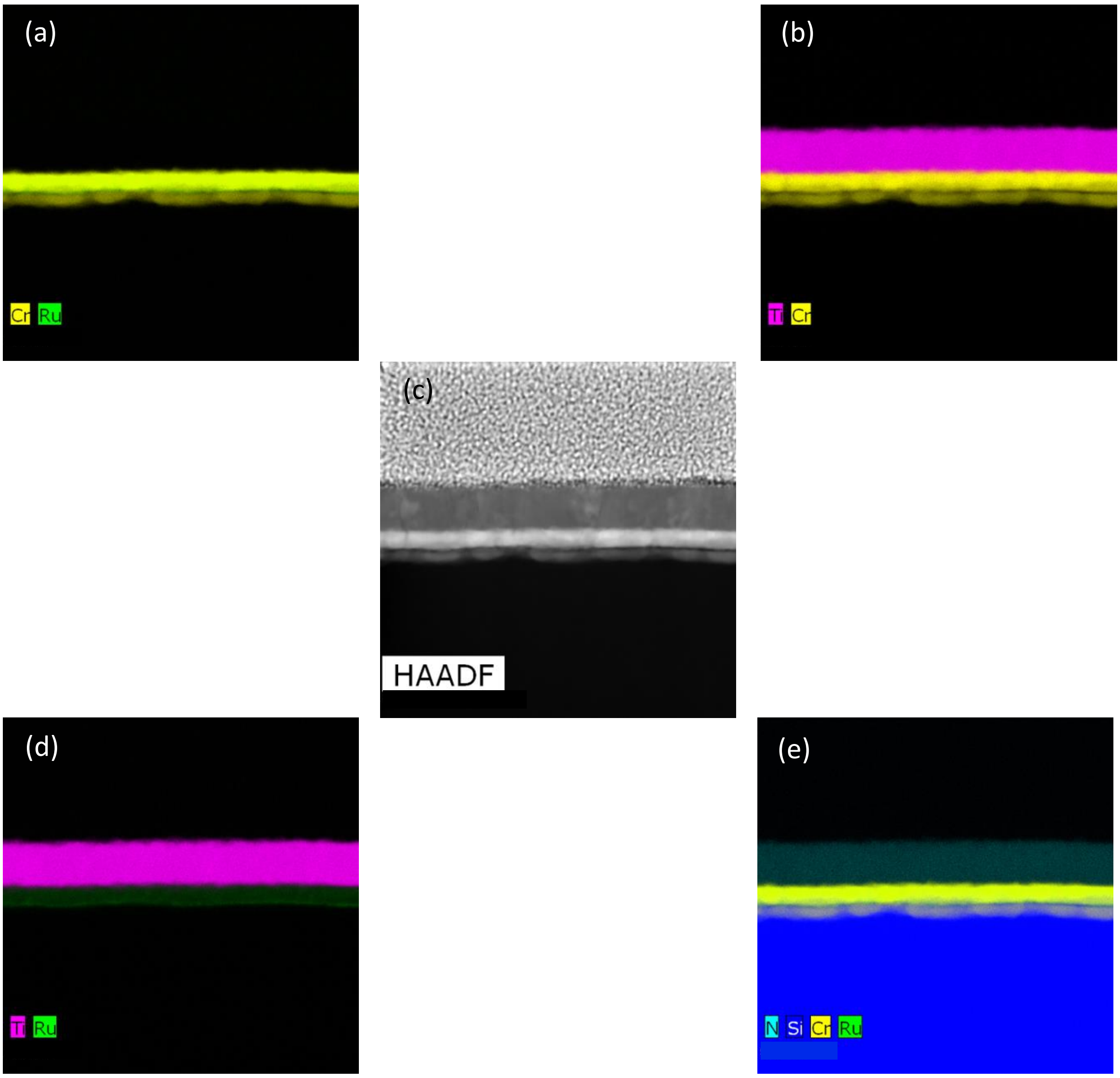

Figure S4: TEM EDX images were acquired using a FEI QUANTAX operating at energy 200 kV equipped with a Bruker Espirit detector. The TiN layer of 50 nm is overgrown on top of a 25 nm CrRu seed layer. (a) shows CrRu layer, (b) shows the Ti and Cr layers, (c) us a HAADF image of the layer stack with a Pt protective coating, (d) shows Ti and Ru layers and lastly (e) shows the substrate (Si) with CrRu layers as well as the N content of TiN layer.

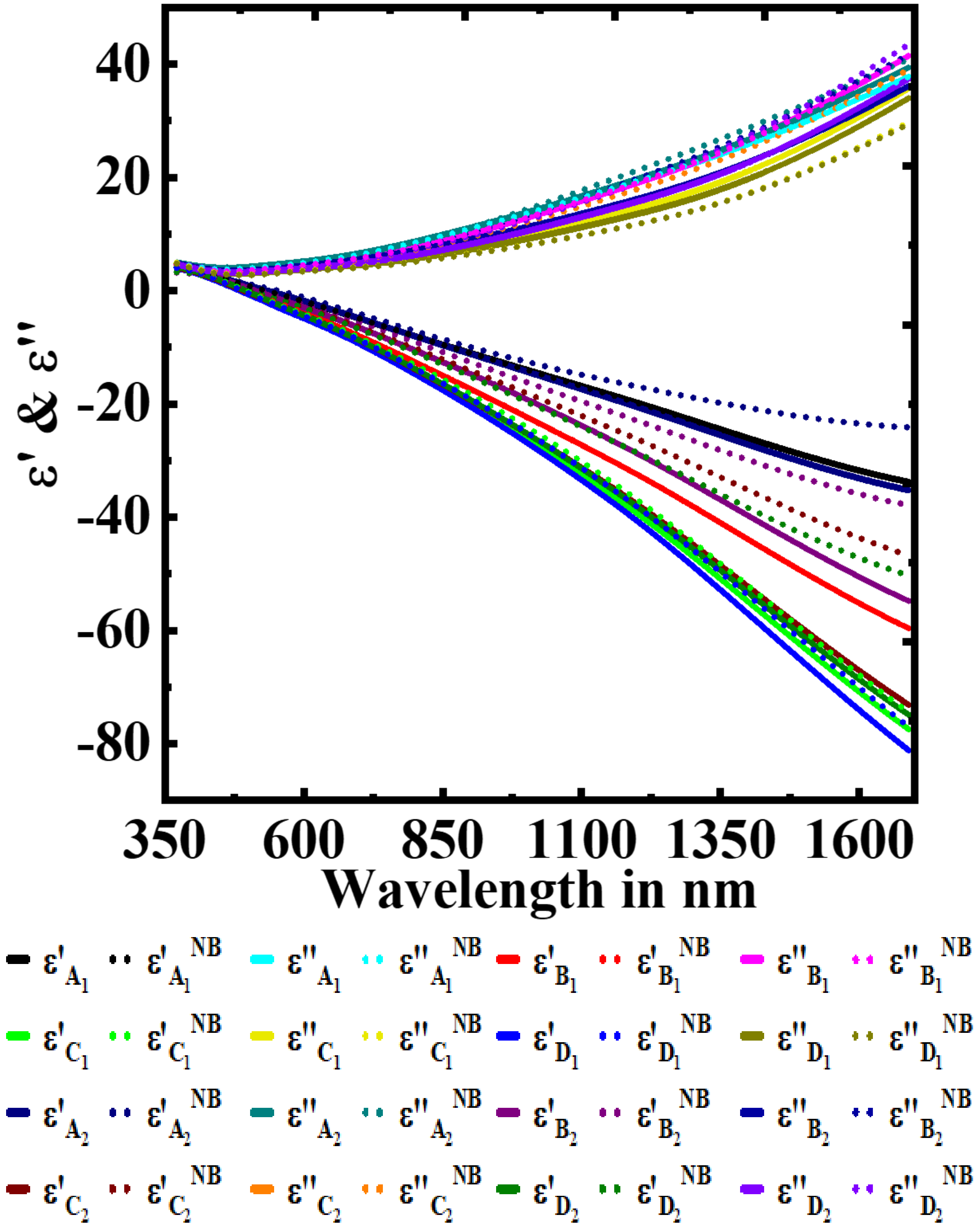

Figure S5: Dielectric functions of all the TiN thin films deposited at different temperatures and partial pressures.

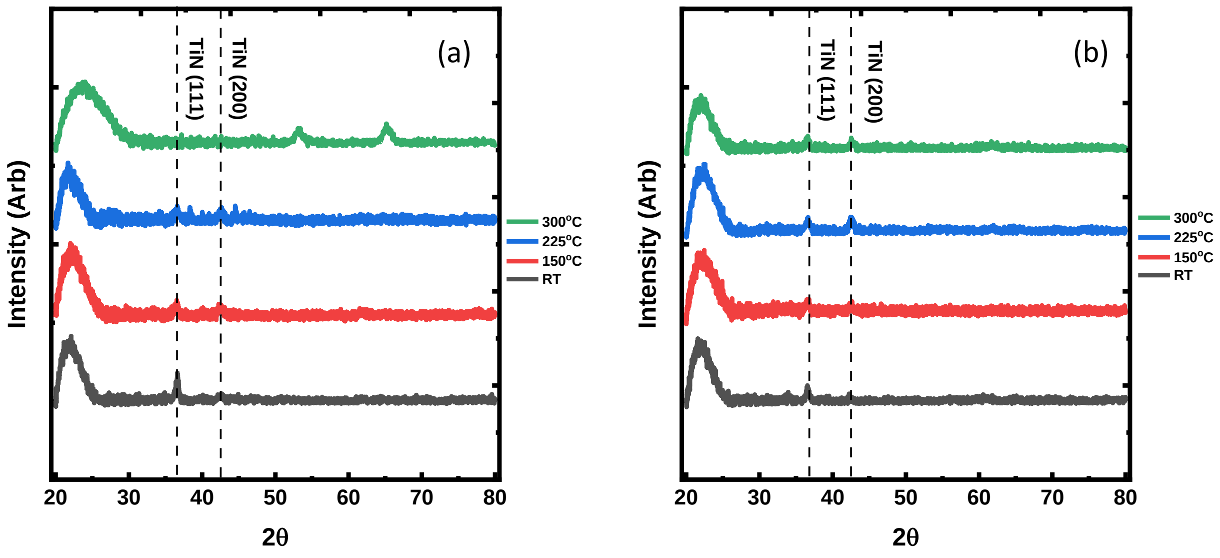

Figure S6: XRD scans of TiN samples deposited at P_1 (a) and P_2 (b) with no buffer.

(a)

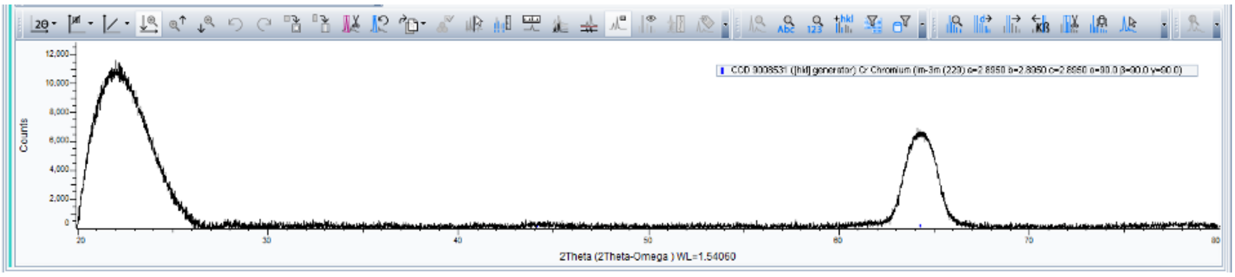

(b)

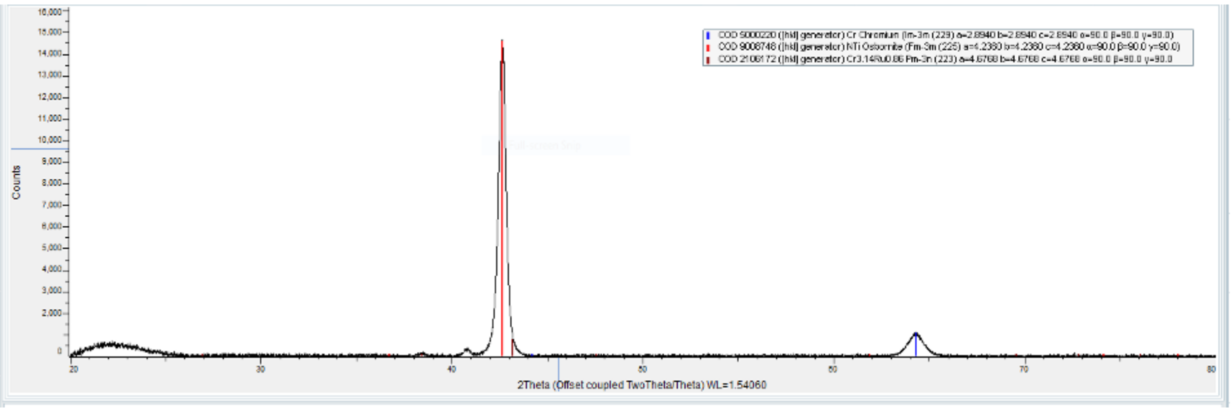

Figure S7**:** Screenshot of the Diffrac.eva XRD modeling software by Bruker™ utilized for peak fitting analysis on the $Cr_{90}Ru_{10}$ sample (a). The software reveals crucial insights into the crystal structure, demonstrating a cubic configuration in a (200) direction, characterized by a lattice parameter of a=2.895Å

The bottom screenshot (b) reveals the analysis of TiN sample on top of a CrRu buffer layer. Fitting of the peaks to the database shows two possibilities for CrRu layer to be. Both grow in the same (200) direction, but they have different lattice parameters. COD5000220 for Cr shows that the lattice parameter is a=2.8940Å for a cubic structure. The COD2106172 for CrRu which fits to the peak at 38.46° has a lattice parameter of a=4.6768Å and the same growth direction of (200).

**Vacuum Anneal Temp (ºC)**	**Oxide (nm)**	**ATM Anneal Temperature (ºC)**	**Oxide (nm)**
Pre-anneal	0.87	Pre-anneal	0.87
300	0.87	300	1.31
400	0.98	400	4.19
500	0.94	X	X
600	0.95	X	X

Table S1: The oxidisation effect on top of TiN thin film at different annealing studies. The oxide layer variance for annealing in the atmospheric condition is much larger than annealing it in vacuum. The oxide layer measurements have been done using XRR technique and it has an attributed error value of ± 1nm.

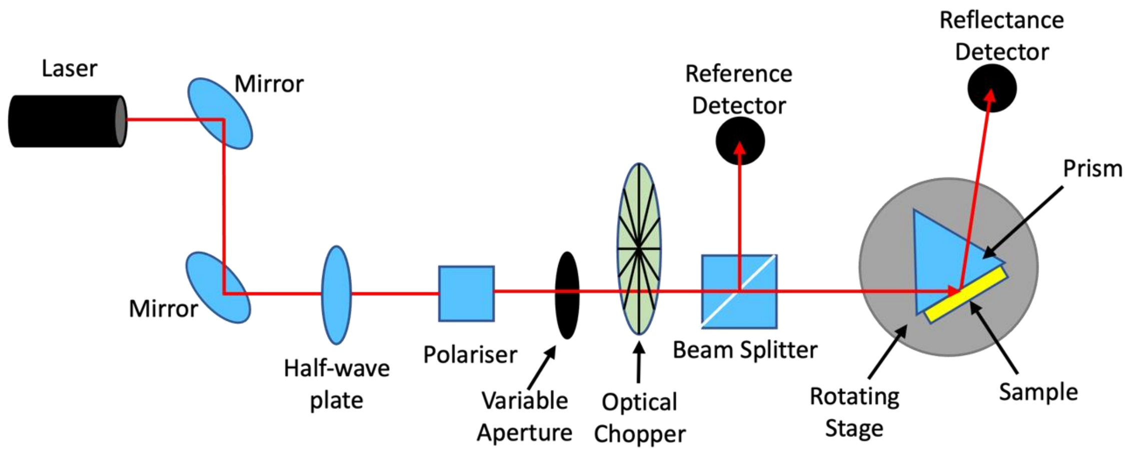

Figure S9: Schematic diagram of the experimental setup of the ATR in Kretschmann configuration

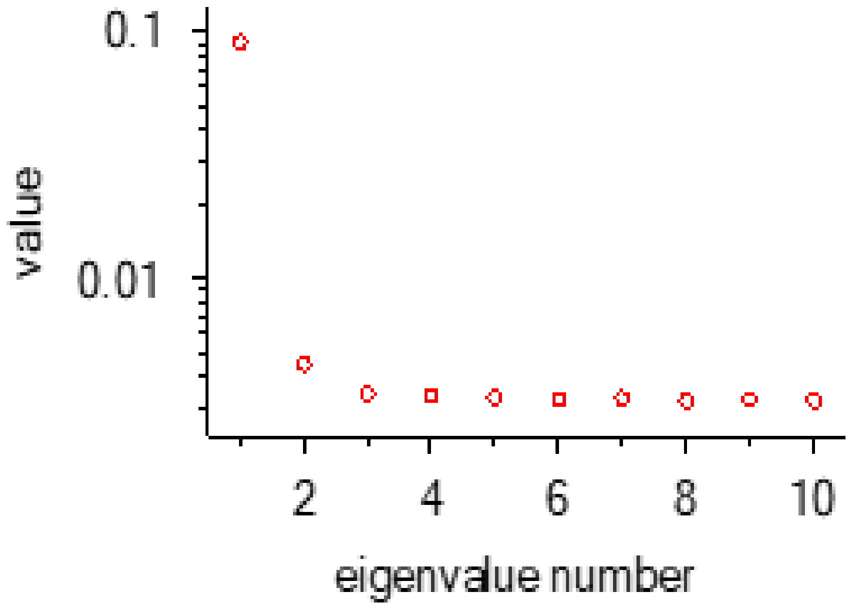

Figure S10: Scree plot of the extracted eigenvalues for the first 10 principle principal components of the hyperspectral CL image. Values represent the fraction of the variance of the full dataset, which is contributed by each component, ordered by decreasing contribution. By inspection, only 1 component stand out.
